# Late complications of robot-assisted radical cystectomy with totally intracorporeal urinary diversion

**DOI:** 10.1007/s00345-020-03378-7

**Published:** 2020-08-03

**Authors:** F. Presicce, C. Leonardo, G. Tuderti, A. Brassetti, R. Mastroianni, A. Bove, L. Misuraca, U. Anceschi, M. Ferriero, M. Gallucci, G. Simone

**Affiliations:** 1 Department of Urology, Sapienza - University of Rome, Via Alessandro Torlonia 12, Rome, Italy; 2 Department of Urology, Regina Elena National Cancer Institute, Rome, Italy

**Keywords:** Robotic cystectomy, Complications, Intracorporeal urinary diversion, Bladder cancer, Follow-up

## Abstract

**Introduction and objectives:**

To evaluate late complications in a large cohort of patients undergoing robot-assisted radical cystectomy (RARC) with totally intracorporeal urinary diversion (ICUD).

**Materials and methods:**

We prospectively enrolled patients who underwent RARC and ICUD between August 2012 and June 2019. We excluded patients with Ejection fraction < 36%, retinal vasculopathy, ventriculoperitoneal shunts, and those treated without curative intent. All complications and their onset date have been recorded, defined, and graded according to Clavien classification adapted for radical cystectomy.

**Results:**

210 patients were included, 76% of whom were men, with a mean age of 62 years. Urinary diversions used were Padua Ileal Bladder (PIB) in 80% of cases, and ileal conduit (IC) in 20% of patients (generally older and with more comorbidity). The mean follow-up was 30 ± 22 months. The stenosis rate of uretero-ileal anastomosis was 14%, while a reduction in eGFR (≥ 20%) was observed in about half of the cases. UTIs occurred in 37% of the patients, especially in the first 12 months. Only 2% of patients had bowel occlusion, whereas incisional hernia, lymphocele, and systemic events (metabolic acidosis and major cardiovascular events) occurred respectively in 20%, 10%, and 1% of cases.

**Conclusions:**

Our study evaluates first late complications in a cohort of patients who underwent RARC with ICUD. These data are encouraging and in line with findings from a historical series of open radical cystectomy (ORC). This study is a further step in supporting RARC as a safe and effective surgical option for the treatment of muscle-invasive bladder cancer (MIBC) in tertiary referral centers.

**Electronic supplementary material:**

The online version of this article (10.1007/s00345-020-03378-7) contains supplementary material, which is available to authorized users.

## Introduction

Open radical cystectomy (ORC) is still the standard treatment for MIBC and recurrent high-grade NMIBC [1]. However, over the last decade, robot-assisted radical cystectomy (RARC) has gradually gained popularity as a possible alternative therapeutic option [1]. In recent studies RARC has shown lower rate of intra- and peri-operative complications, with oncological results comparable to ORC, but with a longer operative time. In fact, recent evidence in the literature shows that RARC compared to ORC allows reduced blood losses and a shorter post-operative stay [2]. Furthermore, RARC has a lower rate of severe postoperative complications in the first ninety days of surgery [2]. Lastly, RARC would seem to provide oncological results comparable to ORC, at least in the short and medium-term [3–5].

However, although there is an increasing interest in RARC and several publications that have been published on this topic in recent years, it is not yet possible to draw definitive conclusions on the advantages and possible limitations of the robotic approach with respect to the open technique [1–5].

In fact, most of the data currently available derive from single-center series, with extracorporeal urinary derivation (ECUD) and with limited follow-up periods [1].

Furthermore, as also mentioned in the guidelines of the European Society of Urology (EAU), there are no available studies on late complications of RARC performed with totally intracorporeal urinary derivations (ICUD) [1].

Aim of our study was to evaluate, for the first time in the literature, late complications in a large cohort of patients undergoing RARC with totally intracorporeal urinary diversion (ICUD).

## Materials and methods

### Design of the study

We prospectively enrolled patients undergoing RARC and ICUD between August 2012 and June 2019.

All the data were inserted prospectively in a database approved by the institutional ethics committee and then retrospectively analyzed.

Inclusion criteria were muscle-invasive urothelial carcinoma of the bladder or recurrent high-grade non-muscle-invasive bladder cancer refractory to intravesical immunotherapy.

Severe cardiovascular diseases with an ejection fraction < 36%, retinal vasculopathy and the presence of a ventriculoperitoneal shunt were the only contraindications to robotic surgery.

Patients treated for non-curative purposes (cT4b, palliative or salvage cystectomy, recurrent disease after radiotherapy) were also excluded from the analysis.

Demographic characteristics of the patients at the baseline, clinical, perioperative, pathological, and functional data and the short and long-term complications were systematically collected.

### Surgical technique, perioperative assistance, and pathological assessment

A rigorous multidisciplinary approach has been employed towards all patients with BCa, which has led to a substantial increase in the use of neoadjuvant chemotherapy.

ERAS (Enhanced Recovery After Surgery) protocols have also been adopted since the end of 2012 [6].

RARC with totally intracorporeal urinary diversion was performed with a surgical technique replicating the principles of the open approach and was extensively described in previous works of our group [3].

### Follow-up schedule

Follow-up was performed in accordance with the guidelines of the European Association of Urology (EAU) [1].

To exclude a recurrence of local and distant disease, urinary cytology and a chest/abdomen CT were performed every six months up to the third year and then annually thereafter.

During follow-up, a cystoscopy and/or a PET/CT scan may have been requested at the discretion of the physicians.

Periodically, an estimate of the glomerular filtration rate (eGFR) according to the CKD-EPI (Chronic Kidney Disease Epidemiology Collaboration) formula was finally recorded for each patient [7].

### Complications

All complications and their onset date have been recorded, defined, and graded according to the Clavien classification based on 5 degrees and 11 domains adapted for radical cystectomy [8]. The Clavien system is, in fact, generally used to report complications in a defined interval, usually ninety days, for perioperative complications. Possible complications were collected at each follow-up visit and all patients enrolled in the study had completed monitoring for at least 90 days after surgery, as we considered all those that occurred after 90 days from surgery as late complications.

### Statistical analysis

Medians with interquartile ranges and means with standard deviations were used to report continuous variables, frequencies, and proportions for categorical variables.

Kaplan–Meier method was used to plot survival curves; survival rates were computed at 1, 2 and 5 years after surgery. The significance level was set to a *p*-value < 0.05. The statistical analysis was performed using the statistical package for Social Science (SPSS v. 24.0; IBM, Somers, NY, USA).

## Results

Overall, 210 consecutive patients were included. Most were male patients (76%), with a mean age of 61 ± 8.7 years and a BMI of 25.6 ± 3.7 kg/m^2^. Neoadjuvant chemotherapy was performed in 26% of the cases and the mean number of lymph nodes (LN) removed for the procedure was 30 ± 12. The most commonly used urinary diversion was PIB (80% of the cases), while the remaining patients underwent IC (20%). At baseline, 16% of patients had grade 3 or higher chronic kidney disease (CKD ≥ 3), while pre-operative mean eGFR value in the enrolled population was 82 ml/min (Table 1).

**Table 1:** Characteristics of the study population at baseline*

Age (years)	61 ± 8.68
Gender (males/females)	160 (76%)/50 (24%)
BMI (kg/m^2^)	25.6 ± 3.7
Diabetes	25/210 (12%)
Hypertension	77/210 (37%)
Myocardial infarction	24/210 (11%)
Hydronephrosis	33/210 (18%)
Preoperative eGFR	82 ± 24
Preoperative creatinine (mg/dl)	1.03 ± 0.4
Preoperative CKD ≥ 3	33/210 (16%)
Derivation	
Ileal conduit	43/210 (20%)
Padua ileal bladder	167/210 (80%)
American Society Anaesthesiology (ASA) score	
1	27/210 (12%)
2	147/210 (68%)
3	36/210 (20%)
4	0/210 (0%)
Surgical indication	
BCG Failure**	120/210 (57%)
Primary MIBC	90/210 (43%)
Neoadjuvant chemotherapy	54/172*** (31%)
Stage pTNM	
≥ T3a	59/210 (21%)
pN +	52/210 (25%)
Lymph nodes removed	30 ± 12
Positive surgical margins	19/210 (9%)
Operative time (minutes)	315 ± 71.2
Preoperative haemoglobin (gr/dl)	11.6 ± 1.8
24 h Post-operative haemoglobin (gr/dl)	10.3 ± 1.3
Time to bowel (days)	4.4 ± 1.9
Hospital stay (days)	12.6 ± 10

*MIBC* muscle-invasive bladder cancer, *NMIBC* not MIBC

*Data are reported as mean value ± SD and *n* (%) respectively for continuous and categorical variables

**BCG failure category includes patients with recurrent high-grade NMIBC and patients with MIBC progression during follow-up

***Number of patients with cT2-T4a, cN0M0 bladder cancer potentially eligible for neoadjuvant chemotherapy

Patients with IC compared to those who received a PIB were generally older (64.7 ± 9.6 vs 60.6 ± 8.2 years), with greater comorbidities, with worse renal function (CKD ≥ 3 in 24% vs 14% of cases) and a more advanced disease stage (disease stage ≥ pT3a in 36% vs 26% of cases) (Table 2).

**Table 2:** Characteristics of the study population at baseline according to the type of urinary derivation performed*

	IC	PIB	*p*
Numbers	43	167	
Age (years)	64.7 ± 9.6	60.6 ± 8.2	0.002
Gender (males/females)	72%/28%	75%/25%	0.700
BMI (kg/m^2^)	26 ± 3	26 ± 4	0.465
Diabetes	7/43 (16%)	18/167 (11%)	0.001
Hypertension	17/43 (40%)	60/167 (36%)	0.730
Myocardial infarction	7/43 (16%)	17/167 (10%)	0.001
Hydronephrosis	8/43 (18%)	25/167 (15%)	0.001
American Society.Anaesthesiology (ASA) score			
1	5/43 (12%)	22/167 (13%)	0.456
2	29/43 (68%)	118/167 (71%)	
3	9/43 (20%)	27/167 (16%)	
4	0/43 (0%)	0/167 (0%)	
Neoadjuvant chemotherapy	14/43 (34%)	40/167 (24%)	0.001
Stage pTNM			
≥ T3a	16/43 (37%)	43/167 (26%)	0.001
pN +	22/43 (50%)	30/167 (18%)	0.001
Operative time (minutes)	299 ± 70	320 ± 71	0.137
Preoperative haemoglobin (gr/dl)	14.5 ± 2.3	13.9 ± 1.9	0.191
24 h post-operative haemoglobin (gr/dl)	11.1 ± 3.5	10.5 ± 2.5	0.576
Time to bowel (days)	4.4 ± 1.9	4.7 ± 2.1	0.234
Hospital stay (days)	10.6 ± 3	13.1 ± 12	0.873
Positive surgical margins	9/43 (21%)	10/167 (6%)	0.001
Preoperative eGFR	79.9 ± 23	82.9 ± 24	0.002
Preoperative CKD ≥ 3	10/43 (24%)	23/167 (14%)	0.001

*MIBC* muscle invasive bladder cancer, *NMIBC* not MIBC

*Data are reported as mean value ± SD and *n* (%) respectively for continuous and categorical variables

Mean follow-up was 30 ± 22 months, with about half of patients reaching at least 2 years of follow-up (Supplementary Fig. S1).

At 5 years, recurrence-free survival (RFS), cancer-specific survival (CSS) and overall survival (OS) were 57 ± 5%, 59 ± 5% and 57 ± 5, respectively % (Supplementary Figs. S2–S4).

### Complications

#### Safety of the upper urinary tract

During follow-up approximately 14% of patients experienced a stenosis of uretero-ileal anastomosis, without statistically significant differences between the two diversions (12% vs 15% for IC and PIB, respectively). Despite the low rate of stenosis, a significant decline in eGFR was observed, in particular for patients who underwent IC.

Likewise, an increase in the rate of patients with a CKD ≥ 3 was observed (CKD ≥ 3 in 44% and 58% of patients underwent PIB and IC, respectively; Table 3).

**Table 3:** List of complications in the study population and relative incidences

Complications	Total (210 pts)	IC (43 pts)	PIB (167 pts)	*p*
Lymphocele	19 (9%)	7 (16%)	12 (7%)	0.113
Incisional hernia	42 (20%)	7 (16%)	35 (21%)	0.640
Stones	17 (8%)	0 (0%)	17 (10%)	0.047
Ureteroileal stricture	30 (14%)	5 (12%)	25 (15%)	0.658
UTI	78 (37%)	15 (34%)	63 (38%)	0.312
Cardiovascular events	2 (1%)	0 (0%)	2 (1%)	0.956
Metabolic acidosis	3 (1%)	1 (2%)	2 (1%)	0.435
Bowel occlusion	4 (2%)	2 (4%)	2 (1%)	0.435
10% eGFR reduction	149 (71%)	37(87%)	112 (67%)	0.001
20% eGFR reduction	109 (52%)	34 (79%)	75 (45%)	0.001
CKD ≥ 3a	98 (47%)	25 (58%)	73 (44%)	0.001

#### Stones and infective complications

None of the patients with IC developed urinary stones compared to 8% of PIB group.

The distribution of the events was quite homogeneous over the years (Tables 3, Supplementary Table S4).

Symptomatic urinary tract infection has been recorded in approximately 37% of cases during follow-up, without statistically significant differences between the two diversions.

The vast majority of the events were recorded in the first year of follow-up (Table 3, Supplementary Table S4).

#### Incisional hernia and gastrointestinal complications

Bowel occlusion was a rare event (2%), occurring exclusively during the first year of follow-up (Tables 3, Supplementary Table S4).

In about 20% of cases, an incisional hernia was observed, without statistically significant differences between the two diversions (Tables 3, Supplementary Table S4).

### Systemic events

Metabolic acidosis and acute cardiovascular events were particularly rare (about 1%) and observed exclusively during the first year of follow-up (Tables 3, Supplementary Table S4).

In almost 10% of patients a lymphocele has been reported, occurring mainly during the first 12 months after surgery (Tables 3, Supplementary Table SS4).

## Discussion

It has now been 16 years since Menon et al. have described RARC procedure for the first time but it is especially in the last decade that this approach has become more widespread throughout the world [9].

At the same time, publications on this topic have also increased. In particular, numerous evidences demonstrated equivalence between RARC and ORC in terms of oncological results not only in the short term but also in the medium-long term (5 years of follow-up) [1–5, 10].

Moreover, RARC is a safe procedure, with a complication rate in the first 30 and 90 days equal to or less than ORC, regardless of whether to perform a totally intracorporeal or extra urinary diversion [11]. Conversely, data on late complications of RARC have been lacking in the literature until now [1, 12].

The reason is probably twofold: first of all, RARC has been running for a short time and with low volumes in many centers, therefore, a sample size with adequate follow-up has not yet been reached to try to answer this question; moreover, there is probably an intrinsic difficulty in systematically collecting late complications, considering that even for the ORC there are not many data on this topic in the literature.

In fact, Clavien system has proved to be a very useful and practical tool for classifying and grading perioperative complications in a short, well-defined time interval of 30 or 90 days after surgery [8, 13].

Unfortunately, this system is less suitable for reporting late complications. This is because some complications can develop, as demonstrated by our results (Supplementary Table S4), in a time-dependent manner.

Therefore, it is important to keep this concept in mind because otherwise early complications are over-represented while late ones could be underrepresented.

To the best of our knowledge, this is the first study to report late complications in the medium term (about 30 months of follow-up) in a consecutive series of patients who underwent RARC with ICUD.

The results appear to be largely in line with the previous literature from open series on this topic.

In the Hautmann report, at 30 months the stenosis rate of ureteroileal anastomosis in patients who underwent neobladder surgery ranged between 5 and 15%, depending on the anastomotic technique adopted [14].

Similarly, in the Studer series including more than 400 patients who underwent ORC with IC, the stricture rate of ureteroileal anastomosis was around 14% with a 100 months follow-up [15].

In our series, the ureteroileal stricture rate, using the split nipple technique, was 15% for PIB and 12% for IC with a mean follow-up of 30 months. Longer follow-up is required to properly assess the risk of developing stricture over time.

Renal function evaluated as eGFR also tends to decrease significantly over time both in the group of patients subjected to PIB and in those with a IC.

In our experience, in fact, a 10% decline in eGFR was observed in about 70% of patients and a 20% eGFR reduction in almost 50% of the cohort.

Similarly, Gershman et al., in a series of about 1300 patients who underwent ORC, observed an eGFR decline of 10% in about 73% of cases and 20% eGFR decline in 61% of patients at 5 years [16].

The rate of urinary tract infections (UTIs) in patients undergoing cystectomy is very variable depending on the parameter used to define them.

In the Studer series, only including UTIs requiring hospitalization, the percentage was 23% [15]. In the Hautmann cohort, recording UTIs associated with fever, the incidence was around 5% [14]. In Wood series, including about 70 ORC and neo-bladder patients, 78% of asymptomatic bacteriuria and 8% of urosepsis were found, respectively [17]. In our cohort, 37% of symptomatic UTIs were recorded and, as in the open series, many events occurred early in the course of follow-up.

No conduit stones were recorded in patients who underwent IC in our study.

Similarly, Studer series had not reported any events in the first two years of follow-up, but the percentage rose to 20% after 5 years from the surgical procedure [15].

According to this time trend, 10% of our patients who underwent PIB experienced stones of the reservoir with a more marked increase in cases starting from the third year of follow-up.

The incidence of bowel occlusion after ORC was in the previous literature between 0.8 and 11% [14, 18, 19]. In our series, we recorded a very low rate of bowel obstruction (around 2%). These findings could be probably explained by the fact that a totally intracorporeal reconstruction, by limitedly mobilizing intestinal loops, can reduce the occlusive risk to a minimum.

Finally, in line with the literature from open series, the cases of severe metabolic acidosis were around 1% and above all in the first year of follow-up [14, 15, 18, 19].

In light of these results and from comparisons with the open series, our study first demonstrates that RARC with ICUD stands the test of time, providing sufficient quality standards in terms of late complications.

However, some limitations of our study should be acknowledged. First, a follow-up time of about 30 months, although of a certain temporal extension, may not be sufficient to intercept all late complications. In fact, as described in the literature, some complications are time-dependent and tend to occur even after a long time from the surgical procedure [14, 15]. Therefore, a prospective systematic collection of complications is mandatory to assess any significant deviations from these preliminary findings in a longer follow-up interval. Furthermore, these data were collected in a tertiary center, with a high surgical volume, with a great surgical experience both with open and robotic procedures, proposing orthotopic neobladder in all cases in which there was no absolute contraindication. Consequently, these results could differ and not be applicable in centers with low surgical volume, with less robotic experience and with other selection criteria for RARC with orthotopic ICUD [20]. This bias is consistent with the wide use of ECUD in most actual RARC series. As a consequence, the two randomized controlled trials comparing open vs RARC only included ECUD [21, 22]. We would aim to provide more definitive outcomes of open RC vs RARC-ICUD with the ongoing randomized clinical trial (NCT03434132) at our Institution.

## Conclusions

We first reported the incidence of late complications following RARC with ICUD. This procedure demonstrated to stand the test of time, being the risk of developing late complications in line with the historical series of ORC. Recent literature has already demonstrated the non-inferiority of RARC in terms of oncological and perioperative results. Therefore, our study is a further step in supporting RARC as safe and effective surgical option for the treatment of MIBC in tertiary referral centers.

## Electronic supplementary material

Below is the link to the electronic supplementary material.

Supplementary Fig. S1 (DOCX 56 kb)

Supplementary Fig. S2 (DOCX 69 kb)

Supplementary Fig. S3 (DOCX 68 kb)

Supplementary Fig. S4 (DOCX 69 kb)

Supplementary Table S4 (DOCX 42 kb)
