# Supplementary figures and images for: Late complications of robot-assisted radical cystectomy with totally intracorporeal urinary diversion
Source: World J Urol. 2020 Aug 3;39(6):1903–9. doi: 10.1007/s00345-020-03378-7 (PMC8217047; doi:10.1007/s00345-020-03378-7)

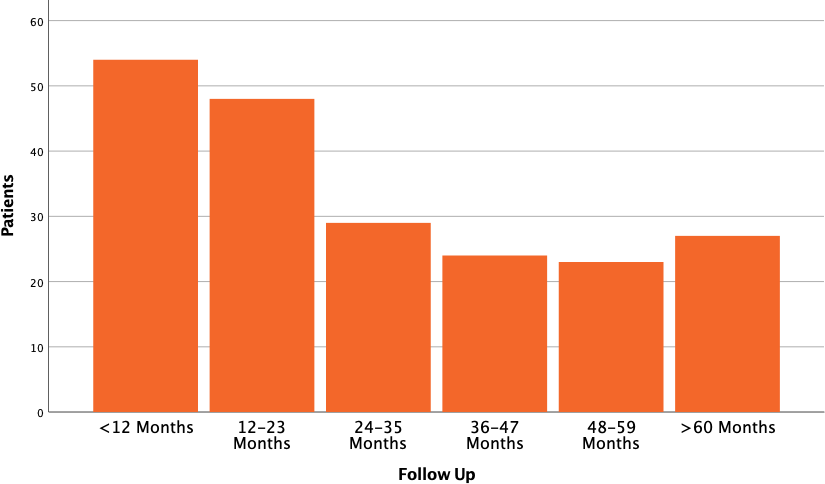
**Figure 1.** **Duration of follow-up in the study population**

Supplement: Supplementary file 1 — Supplementary Fig. S1 (DOCX 56 kb) [file 345_2020_3378_MOESM1_ESM.docx]
