# Supplementary material for: Late complications of robot-assisted radical cystectomy with totally intracorporeal urinary diversion
Source: World J Urol. 2020 Aug 3;39(6):1903–9. doi: 10.1007/s00345-020-03378-7 (PMC8217047; doi:10.1007/s00345-020-03378-7)
Supplement: Supplementary file 4 — Supplementary Fig. S4 (DOCX 69 kb) [file 345_2020_3378_MOESM4_ESM.docx]

**Figure 4. Kaplan-Meier analysis for overall survival in patients undergoing RARC plus ICUD**

**Overall Survival Function**


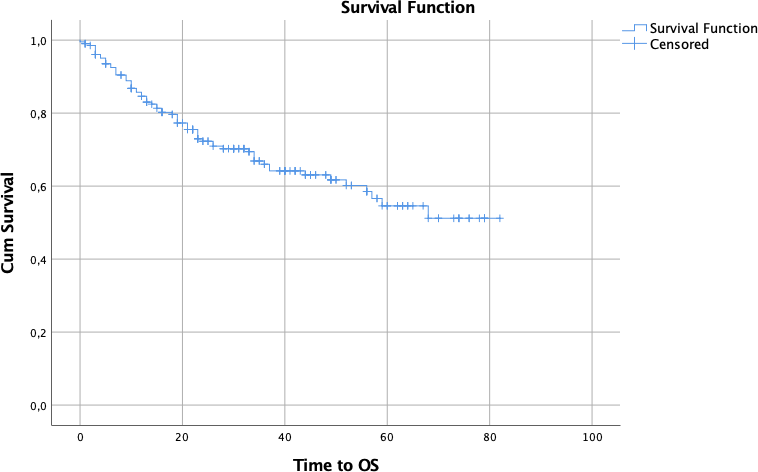


**Cumulative Survival**

**Follow-up Length (Months)**
