# Supplementary material for: Late complications of robot-assisted radical cystectomy with totally intracorporeal urinary diversion
Source: World J Urol. 2020 Aug 3;39(6):1903–9. doi: 10.1007/s00345-020-03378-7 (PMC8217047; doi:10.1007/s00345-020-03378-7)
Supplement: Supplementary file 5 — Supplementary Table S4 (DOCX 42 kb) [file 345_2020_3378_MOESM5_ESM.docx]

**Table 4.** Distribution of complications over time

| **COMPLICATIONS** | **<12 Months** | **12-24 Months** | **>24 Months** |
| --- | --- | --- | --- |
| **Lymphocele** | 13 | 4 | 2 |
| **Incisional Hernia** | 28 | 8 | 6 |
| **Stones** | 5 | 4 | 8 |
| **Ureteroileal stricture** | 15 | 9 | 6 |
| **UTI** | 60 | 7 | 11 |
| **Cardiovascular Events** | 2 | 0 | 0 |
| **Metabolic Acidosis** | 3 | 0 | 0 |
| **Bowel Occlusion** | 4 | 0 | 0 |
